# Vitamin D food fortification in European countries: the underused potential to prevent cancer deaths

**DOI:** 10.1007/s10654-022-00867-4

**Published:** 2022-05-06

**Authors:** Tobias Niedermaier, Thomas Gredner, Sabine Kuznia, Ben Schöttker, Ute Mons, Jeroen Lakerveld, Wolfgang Ahrens, Hermann Brenner, On behalf of the PEN-Consortium

**Affiliations:** 1 Division of Clinical Epidemiology and Aging Research, German Cancer Research Center (DKFZ), Heidelberg, Germany; 2 Medical Faculty Heidelberg, University of Heidelberg, Heidelberg, Germany; 3 Network Aging Research (NAR), University of Heidelberg, Heidelberg, Germany; 4 Department of Epidemiology and Data Science, Amsterdam UMC, Amsterdam, Netherlands; 5 Department of Epidemiological Methods and Etiological Research, Leibniz Institute for Prevention Research and Epidemiology - BIPS, Bremen, Germany; 6 Institute of Statistics, Faculty of Mathematics and Computer Science, University of Bremen, Bremen, Germany; 7 Division of Preventive Oncology, German Cancer Research Center (DKFZ) and National Center for Tumor Diseases (NCT), Heidelberg, Germany; 8 Faculty of Medicine and University Hospital Cologne, University of Cologne, Cologne, Germany; 9 Cancer Prevention Unit, German Cancer Research Center (DKFZ), Heidelberg, Germany; 10 German Cancer Consortium (DKTK), German Cancer Research Center (DKFZ), Heidelberg, Germany

**Keywords:** Vitamin D, Fortification, Cancer mortality, Prevention

## Abstract

*Background*: Meta-analyses of randomized controlled trials have shown that vitamin D supplementation reduces cancer mortality by 13%. Vitamin D fortification of foods may increase vitamin D levels in a similar manner as vitamin D supplementation and could achieve similar reductions in cancer mortality. Whereas some European countries already implemented widespread fortification of foods with vitamin D, in other countries only few or no foods are fortified. In this study, we estimated the reduction in cancer mortality presumably already achieved by current fortification policies in 2017 and the potential for further reductions if all countries had effective fortification. *Methods*: We reviewed scientific literature, publicly available information, and contacted health authorities to obtain information on current vitamin D food fortification policies in 34 European countries. Together with country-specific cancer death statistics from Eurostat, information on life expectancy, and country-specific fortification policies, we used data from studies on supplementation and serum 25(OH)D increases and cancer mortality to estimate numbers of probably already prevented cancer deaths and numbers of potentially further preventable deaths and years of life lost. *Results*: Current vitamin D fortification is estimated to prevent approximately 11,000 in the European Union and 27,000 cancer deaths in all European countries considered per year. If all countries considered here would implement adequate vitamin D fortification of foods, an estimated additional 129,000 cancer deaths (113,000 in the European Union) could be prevented, corresponding to almost 1.2 million prevented years of life lost (1.0 million in the EU) or approximately 9% of cancer deaths (10% in the EU). *Interpretation*: Systematic fortification of foods might considerably reduce the burden of cancer deaths in Europe.

**Supplementary Information:**

The online version contains supplementary material available at 10.1007/s10654-022-00867-4.

## Introduction

Vitamin D is an essential hormone for bone metabolism [1] and involved in many other physiological processes. Recent meta-analyses of randomized clinical trials (RCTs) have rather consistently shown that daily supplementation with vitamin D (with 400–2000 international units (IU) per day) lowers cancer mortality (but not cancer incidence) by approximately 13% in older adults [2], even though individual studies failed to reach statistical significance. In line with this, meta-analyses of observational studies found better survival in cancer patients for a number of cancers, including colorectal, breast, prostate, and pancreatic cancer [3]. Possible mechanisms include increased apoptosis, antiproliferative effects, and immune modulation [4], as well as involvement in angiogenesis and metastasis [5]. The only study that did not find a decrease in cancer mortality was conducted in New Zealand, where UV radiation is stronger [6] and vitamin D deficiency is uncommon [7], especially when compared to Germany [8] or other European countries [9].

Prevalence of vitamin D insufficiency and deficiency is high in European countries, particularly in the older population and non-western immigrants [10], and is highest in winter and spring due to insufficient vitamin D production in the skin under sun exposure during the winter months. Contrary to expectations, vitamin D deficiency is also highly prevalent in Southern European countries, despite abundant sunshine [11]. Although vitamin D supplementation is widely recommended and practiced to prevent rickets among newborns and infants [12], recommendations on vitamin D supplementation among older adults are inconsistent across countries [13], and only a small fraction of the population takes vitamin D supplements regularly. For example, according to data from the UK Biobank, approximately 4% of adults in the UK reported vitamin D intake from supplements [14]. In Germany, approximately 82% of men and 91% of women do not reach the recommended daily intake of vitamin D [15]. Even among those taking supplements, it is unclear if they ingest adequate amounts of vitamin D. For example, common over-the-counter multivitamin products for children [16] and adults typically contain insufficient amounts of vitamin D.

An alternative and comparably effective [17] way of enhancing vitamin D status is fortification of foods with vitamin D. Various European countries already have policies to fortify several food groups to some extent (see Table 1). Fortification is systematically used in Finland and the UK, and less comprehensively also e.g. in Iceland, Sweden, and Ireland. To our knowledge, no other European country systematically fortifies foods with vitamin D, even though voluntary fortification is in principle allowed (but rarely done) in several other countries, including Germany, Denmark, Austria, and Switzerland.

**Table 1:** Overview of vitamin D fortification policies in European countries

Country	Fortification policy/description	Ref.	Cat
Countries of the European Union (27 countries)			
Austria	No mandatory fortification	[46]	—
Belgium	Mandatory: Margarines and spreadable fats (6–7.5 µg/100 g), voluntary: milk, dairy drinks, milk substitutes, cereals, biscuits, fruit juices, lemonades, cocoa powder, weight-loss products	[47]	−
Bulgaria	“Vitamin D food fortification is not mandatory (…) and there is virtually no fortified foodstuff on the market. Fish consumption is also low and we can assume that for the majority of the population the alimentary vitamin D intake is generally scarce.”	[48]	—
Croatia	“(…) despite the fact that the margarines are vitamin D fortified and some fortified dairy products are available, none of them contributes to [a] high vitamin D share in the diet.”	[49]	−
Cyprus	No mandatory fortification (corr. with Cypriotic Public Health Authority, Sept. 21, 2020)	−	—
Czech Republic	“Moreover, there is no mandatory and only minimal voluntary vitamin D food fortification in the Czech Republic.”	[50]	—
Denmark	Allowed, not mandatory, rarely done	[51]	−
Estonia	Allowed but not mandatory	[52]	−
Finland	Mass fortification of milk, margarine/fat spread; fortification of selected brands for yoghurt, orange juice, plant-based milk, bread, cereals^1^	[53]	++
France	Allowed but not mandatory	[54]	−
Germany	No mandatory fortification + previously largely not allowed	[26]	—
Greece	No fortification policy	[55]	—
Hungary	“Because the typical Hungarian diet contains little vitamin D and food is not fortified, the vast majority of vitamin D is formed in the skin by ultraviolet-B (UVB) radiation.”	[56]	—
Ireland	Voluntary fortification of milk (~ 160 IU/200 ml) and cereals (~ 118 IU/35 g), mandatory fortification of margarine	[57, 58]	o
Italy	“(…) lack of appropriate fortification and supplementation of foods.”	[59]	—
Lithuania	No fortification (corr. with Ministry of Health, Sept. 14, 2020)	−	—
Luxembourg	No fortification (corr. with Ministry of Health, Oct. 20, 2020)		—
Malta	No fortification policy; fortified yoghurt for children available (corr. with Maria Bonnici, Jan. 25, 2021). Since 2021 also fortified milk is available [60]	−	—
The Netherlands	No mandatory fortification; voluntary fortification previously allowed only for certain fats and oils, no more than 0.075 µg/g[61], now apparently also some cereals, dairy products and drinks are fortified with 30–650 IU/100 g [62]	[61, 62]	−
Poland	No fortification (except milk formulas for infants and toddlers)	[63]	—
Portugal	No fortification policy/no mandatory fortification, but some fortified foods such as yoghurts, milk, breakfast cereals, etc. are available in the market	[64, 65]	−
Spain	Fortification is voluntary for all foods except for infant formulas and infant cereals. Moreover, (…) there is a tendency to fortify skimmed and semi‐skimmed milks with vitamin D to reach the initial levels of whole milk; in addition, some commercial products are fortified with vitamin D such as biscuits, yoghurt, margarine, cheese, cereals and juices	[66]	−
Romania	“In Romania dietary sources of vitamin D are scarce and there is no fortification of food with vitamin D.”	[67]	—
Slovakia	No special legislation concerning fortification (vitamin D may be voluntary added to foods) (corr. with Slovakian Public Health Authority, Sept. 22, 2020)	−	—
Slovenia	“(…) dairy and other food products are generally not fortified with vitamin D (…).”	[68]	—
Sweden	Low-fat milk, fermented milk products, margarine	[69]	+
Non-EU countries with available data (6 countries)			
Iceland	Low-fat milk, some imported foods (vegetable oils and cereals)	[70]	+
Norway	Largely prohibited, but voluntary fortification of extra low-fat milk and relatively high intake of cod liver oil and fish oil supplements	[71–73]	+
Serbia	No fortification policy	[74]	—
Switzerland	No mandatory fortification	[46]	—
Turkey	“25(OH)D fortified foods are not common in markets and their prices are higher when compared to the similar group of foods.”	[75]	—
UK	Margarine (not less than 7.05 µg and not more than 8.82 µg of vitamin D), infant formula and foods intended for use in energy-restricted diets, bread (200 IU/100 g), orange beverage (1000 IU/240 ml)	[62]	++

*IU* international units; *Ref.* reference; *Corr.* correspondence; *Cat.* categorization into fortification level: ++ : mandatory fortification with adequate amounts covering adequate range of products; + : wide-spread voluntary fortification but with insufficient amounts or adequate mandatory fortification but with too few products, or other wide-spread sources of vitamin D intake; o: insufficient mandatory fortification plus some voluntary fortification, − : no mandatory fortification but commonly some voluntary fortification of foods; —: no mandatory fortification and in practice also almost no voluntary fortification of foods.; —?: unknown, supposedly no fortification. ^1^Amounts of vitamin D in fortified foods might, however, still be inadequate especially during the winter months, with daily uptakes from fortified foods of ~ 330–440 IU/day [76]. Note: no information could be obtained for Latvia, which was assumed to have no fortification. European countries not considered because of missing Eurostat data on cancer deaths (sorted by population size): Ukraine, Belarus, Moldova, Bosnia, Albania, Kosovo, Montenegro, Monaco, Liechtenstein, San Marino, Vatican City

In this study, we estimated the cancer mortality reduction already achieved by current fortification policies and the potential for further reductions if all countries had adequate fortification.

## Methods

We selected 2017 as reference year for our calculations, because it was the latest year with Eurostat data on cancer mortality available at the time of analysis.

### Vitamin D food fortification policies in European countries

Information on fortification policies was obtained from various sources, including information published in recent scientific articles, web pages of health authorities, and via e-mail requests in English language to health authorities of the respective countries between August and October 2020. The email addresses were identified with the help of Europe Direct [18]. As European countries (“Europe”), we included all EU countries plus European non-EU countries with available Eurostat data on cancer mortality (see Table 2). Food fortification is not prohibited by laws or regulations of the European Union (EU), as long as certain requirements are fulfilled, such as minimum and maximum amounts of nutrients added [19]. Also, it is possible that the extent of food fortification changes over time—also in the absence of clear fortification policies—which makes up-to-date summaries difficult.

**Table 2:** Numbers of cancer deaths in 2017 by age group and sex in Europe and in the European Union according to Eurostat [24]

Age	Men	Women	Total
*Europe*			
50–54	26,677	23,432	58,006
55–59	58,103	40,518	95,977
60–64	88,045	55,865	143,006
65–69	115,234	72,338	186,114
70–74	118,721	77,664	201,641
75–79	132,024	93,660	223,778
80–84	121,166	97,664	218,687
85–89	85,163	83,154	169,457
90–94	34,177	45,080	80,262
≥ 95	6,720	12,499	21,061
Sum	791,543	605,299	1,397,989
*European Union (excluding United Kingdom)*			
50–54	24,007	20,514	45,547
55–59	46,329	32,252	78,581
60–64	69,990	44,758	114,748
65–69	91,351	57,168	148,519
70–74	93,904	60,802	154,706
75–79	106,756	75,058	181,814
80–84	97,488	79,403	176,891
85–89	69,110	68,238	137,348
90–94	27,022	37,229	64,251
≥ 95	5,191	10,079	15,270
Sum	631,889	485,786	1,117,675

Data available from: https://ec.europa.eu/eurostat/data/database → Database by themes → Population and social conditions → Health (hlth) → Causes of death (hlth_cdeath) → General mortality (hlth_cd_gmor) → Causes of death—deaths by country of residence and occurrence (hlth_cd_aro)

Nevertheless, with the exception of one country (Latvia) contributing less than 0.04% to the annual number of cancer deaths in Europe (< 0.06% in the EU), we were able to assign all countries to one of the following categories based on the extent of food fortification currently employed:

- ++ Mandatory fortification with adequate amounts covering an adequate range (four or more commonly consumed foods) of products;
- + Wide-spread voluntary fortification but with insufficient amounts or adequate mandatory fortification of too few products;
- o Insufficient mandatory fortification plus some voluntary fortification,
 − No mandatory fortification but some voluntary fortification of foods;
 −− No mandatory fortification and almost no voluntary fortification of foods.

For Latvia, we assumed that no fortification was used in 2017 because we did not find any evidence for a fortification policy in Latvia. Effects of fortification on cancer mortality have not been studied previously. However, it has been demonstrated that food fortification can elevate serum 25(OH)D levels to an extent comparable to supplementation of ~ 400 IU per day [17], for which cancer mortality reductions by 11% have been found [20]. Furthermore, dose–response relations between intake of vitamin D fortified food and serum 25(OH)D concentrations have been demonstrated [21]. We thus imputed expected effects of fortification policies based on the 11% cancer mortality reduction observed in an RCT with daily supplementation of 400 IU [20]. We assumed a proportionally reduced effect size for lower doses, depending on the currently employed extent of fortification. Currently achieved reduction of cancer mortality by vitamin D fortification was assumed to be 9, 7, 5.5, 2, and 0%, and the complementary potential additional reduction by optimal vitamin D fortification was assumed to be 2, 4, 5.5, 9 and 11% for countries in food fortification categories ++ ,  + , o,  − , and −− , respectively. Those are very conservative estimates and rather reflect lower bounds of achievable effects, because they implicitly assume an achievable upper bound of 11% cancer mortality reduction. In fact, however, studies on supplementation demonstrated stronger reductions with higher daily doses, i.e. 11% with 400 IU [20], 15% with 800 IU [22], and 17% with 2000 IU [23]. Those effects were also achieved e.g. in the US and in the UK where foods were already fortified. Also, we were rather conservative in assumptions regarding countries that already fortify foods, such as UK (“ ++ ” even though only orange juice is strongly fortified). Thus, actual effects of adequate fortification are expected to be stronger. To account for statistical uncertainty in the estimates of cancer mortality reduction, we carried out sensitivity analyses in which the proportionate cancer mortality reduction assumed in the various analyses was multiplied by the upper or lower bound of the corresponding 95% confidence interval in the meta-analysis of RCTs (21% or 4%, respectively), divided by the point estimate (13%) (2).

### Cancer deaths in 2017 by country, age and sex

Numbers of cancer deaths, stratified by sex and in 5-year age groups, were taken from Eurostat [24]. The most recent year with data available for all countries at the time of analysis was 2017. Of note, cancer statistics were not available and thus not used from a few European countries, such as Ukraine and Moldova. These countries, accounting for less than 10% of the European population, were therefore not included in our analyses. We restricted our analyses to cancer deaths occurring above 50 years of age (approximately 95% of cancer deaths in Europe), because the evidence for cancer mortality reduction with vitamin D supplementation is based on RCTs among older adults [20, 22, 23].

### Life expectancies and years of life lost

We extracted overall and sex-specific life expectancies for the various countries from Eurostat and from the WHO (Global Health Observatory data repository) [25]. Life expectancy for men and women was averaged and not calculated by sex because sex-specific information was not available for some countries and because reductions in cancer mortality with vitamin D supplementation had been reported only for men and women combined. Using the life expectancies and the midpoints of age intervals, we estimated the further life expectancy for each 5-year age interval. In age intervals exceeding the life expectancy, we assumed further 2 years of life expectancy. Years of life lost due to cancer deaths were calculated as the product of cancer deaths in a certain age group and further life expectancy for individuals of that age in the general population.

### Cancer mortality reductions, prevented cancer deaths and years of life lost

We estimated the cancer mortality reductions already achieved with current fortification policies (compared to no fortification), and further achievable cancer mortality reductions assuming comprehensive fortification in all countries. Depending on the extent of current food fortification, we estimated that cancer mortality could be further reduced by an additional 2–11%. This estimation was based on a previous study that found serum level increases with fortification for various foods to be comparable to supplementation with 400 IU [17], for which 11% mortality reduction have been demonstrated [20]. To account for the possibility that vitamin D supplement intake may have increased since the RCTs on supplementation and cancer mortality were conducted, we performed additional sensitivity analyses in which we assumed a by 10% lower effectiveness of fortification in reducing cancer mortality. Because our effect estimates were based on the RCTs which reported on (overall) cancer mortality reductions via vitamin D supplementation, we could not estimate cancer site-specific effects. Preventable years of life lost (YLL) were estimated by first calculating the difference between (country-specific) life expectancy and age at cancer death and then multiplying those YLL with the assumed effect estimate of fortification (2–11%).

Based on the observed number of cancer deaths (ON), the expected number of cancer deaths without fortification was calculated as ON/(1–P) where P denotes the assumed current proportional reduction of cancer mortality (assumed to range from 0.00 to 0.09), and the number of already prevented cancer deaths was calculated as (ON/(1–P)) × P. For example, if we assume that a country’s current fortification policy has already reduced cancer mortality by 2% = 0.02, then the assumed number of deaths without fortification would be ON/0.98, and the number of already prevented cancer deaths would be (ON/0.98) × 0.02. The proportion of further preventable cancer deaths was calculated as (ON/(1–P)) × (0.11–P), i.e. by multiplying ON/(1–P), the expected number of cancer deaths without fortification, with the remaining potential additional proportional reduction in cancer mortality by optimal fortification (0.11 − P, assumed to range between 0.02 and 0.11). Fortification has already been implemented in the US and in the UK, and four out of five RCTs included in the meta-analyses on the impact of vitamin D supplementation on cancer mortality were conducted in these two countries.

Calculations were done for EU countries (excluding the UK) and for a number of European non-EU countries reported on in Eurostat (Iceland, Norway, Serbia, Switzerland, Turkey, United Kingdom), hereafter referred to as “Europe” for simplicity. This definition excludes countries for which Eurostat data were unavailable (Ukraine, Belarus, Moldova, Bosnia, Albania, Kosovo, Montenegro, Monaco, Liechtenstein, San Marino, Vatican City). All statistical analyses were done in R version 4.0.5.

## Results

### Vitamin D food fortification policies in European countries

Vitamin D fortification policies for food are summarized in Table 1. Overall, the vast majority of European countries did not appear to have policies in place to adequately fortify a relevant range of foods with vitamin D. Exceptions were Finland and the UK which fortify a number of commonly consumed foods with adequate amounts.

### Cancer deaths in 2017 by age and sex

Numbers of cancer deaths in Europe in 2017, stratified by age and sex, are summarized in Table 2. Overall, approximately 1.4 million individuals aged > 50 years died from cancer in Europe in 2017, thereof more men (approximately 792 million) than women (approximately 605 million). Overall, cancer caused 24% of all deaths. When focusing on the EU, the respective number for 2017 was approximately 1.1 million individuals (again 24% of all deaths), thereof 632,000 men and 486,000 women.

### Life expectancies and years of life lost

For the countries reported on in Eurostat (all EU countries plus most European non-EU countries), life-expectancies were extracted from Eurostat and WHO data. Life expectancies ranged from 70.9 years (Latvia) to 83.7 years (Switzerland) and were consistently higher among females (Supplementary Table 1). An estimated total of 12.6 million years of life were lost in the investigated countries due to cancer deaths in 2017.

### Cancer mortality reductions, prevented cancer deaths and years of life lost

We assumed achievable cancer mortality reductions by 2–11%, depending on the extent of current fortification. With these assumptions, a total of approximately 27,400 cancer deaths has presumably already been prevented in 2017 in Europe (thereof almost 11,000 in the EU excluding the UK), with the potential for prevention of further 129,000 cancer deaths (9% of cancer deaths), thereof 113,000 in the EU (10% of cancer deaths in the EU) if all countries used adequate fortification of foods with vitamin D like Finland (Table 3). This would correspond to approximately 1.2 million preventable years of life lost (approximately 1 million in the EU).

**Table 3:** Estimates of prevented years of life lost in the European population and in the population of the European Union aged 50 years and older in 2017 using different assumptions regarding vitamin D fortification effects

Country	Fortification policy^c^	Annual cancer deaths baseline^d^	Probably currently prevented cancer deaths	Further potentially preventable cancer deaths	Preventable YLL
*European Union*					
Austria	—	19,663	0	2,163	20,579
Belgium	−	25,641	523	2,355	21,259
Bulgaria	—	16,216	0	1,784	12,750
Croatia	−	13,231	270	1,215	9,853
Cyprus	—	1,345	0	148	1,557
Czech Republic	—	26,290	0	2,892	24,980
Denmark	−	15,139	309	1,390	11,884
Estonia	−	3,686	75	338	2,411
Finland	++	12,205	1,207	268	2,409
France	−	156,972	3,204	14,416	141,958
Germany	—	220,462	0	24,251	208,692
Greece	—	28,664	0	3,153	27,468
Hungary	—	31,206	0	3,433	28,145
Ireland	o	8,642	360	630	6,048
Italy	—	164,340	0	18,077	164,262
Latvia	—?	5,695	0	626	3,016
Lithuania	—	7,542	0	830	4,245
Luxembourg	—	1,047	0	115	1,150
Malta	—	912	0	100	1,004
The Netherlands	−	43,323	884	3,979	37,933
Poland	—	95,266	0	10,479	91,314
Portugal	−	26,039	531	2,391	22,367
Romania	—	47,787	0	5,257	41,440
Slovakia	—	13,067	0	1,437	11,821
Slovenia	—	6,148	0	676	6,279
Spain	−	104,179	2,126	9,567	98,019
Sweden	+	22,211	1,293	1,293	10,670
*Non-EU countries*					
Iceland	+	572	33	33	307
Norway	+	10,516	612	612	5,604
Serbia	—	20,387	0	2,243	17,828
Switzerland	—	16,915	0	1,861	18,533
Turkey	—	71,658	0	7,882	80,866
United Kingdom	+ +	161,025	15,926	3,539	29,652
Europe^a^, Main	NA	1,397,898	27,353	129,433 (39,440–209,176)	1,166,303 (359,494–1,907,845)
*Weaker effect (-10%)*		−	Preventable: 116,490 (35,496–188,258)		1,049,673 (323,545–1,717,061)
EU^b^, Main	NA	1,116,916	10,782	113,263 (34,365–182,370)	1,013,513 (312,446–1,655,159)
*Weaker effect (-10%)*		−	Preventable: 101,937 (30,929–164,133)		912,162 (281,201–1,489,643)

^a^Countries considered: Austria, Belgium, Bulgaria, Croatia, Cyprus, Czech Republic, Denmark, Estonia, Finland, France, Germany, Greece, Hungary, Iceland, Ireland, Italy, Latvia, Lithuania, Luxembourg, Malta, Netherlands, Norway, Poland, Portugal, Romania, Serbia, Slovakia, Slovenia, Spain, Sweden, Switzerland, Turkey, United Kingdom. Data taken from Eurostat, which does not comprise data of several non-EU countries such as Ukraine (population: approximately 41.6 million), Bosnia and Herzegovina (population: approx. 3.3 million), and Moldova (population: approx. 2.6 million)

^b^Excluding UK

^c^ ++ : mandatory fortification with adequate amounts covering adequate range of products; + : wide-spread voluntary fortification but with insufficient amounts or adequate mandatory fortification but with too few products; o: insufficient mandatory fortification plus some voluntary fortification, − : no mandatory fortification but commonly some voluntary fortification of foods; —: no mandatory fortification and in practice also almost no voluntary fortification of foods.; —?: unknown, supposedly no fortification

^d^According to Globocan data from 2020

*NA* not applicable; *UK* United Kingdom; *EU* European Union; *YLL* years of life lost

Sensitivity analyses assuming stronger or weaker cancer mortality reduction according to the upper and lower bound of the 95% confidence interval of the respective meta-analysis (2) suggested a wide plausible range for preventable cancer deaths in Europe (approximately 39,000–209,000) and the EU (approximately 34,000–182,000). Likewise, the plausible range for preventable years of life lost was wide (approximately 360,000–1.9 million in Europe, and 312,000–1.7 million in the EU).

In sensitivity analyses, we assumed that the proportion of individuals supplementing vitamin D increased by 10% since the RCTs on supplementation and cancer mortality were implemented, thus, no further mortality reduction would be achievable in these individuals. This would reduce our estimate to about 116,000 preventable cancer deaths in Europe (thereof about 102,000 in the EU) and to approximately 1 million preventable YLL (approximately 900,000 in the EU)*.* The lower and upper bounds of the plausible range of preventable cancer deaths and YLL would analogously be lower by 10% compared to the main scenario.

## Discussion

In this study, we first evaluated the currently used potential of vitamin D food fortification for reducing cancer mortality in Europe. Next, we assessed the remaining potential if all countries employed policies comparable to Finland’s by using widespread and adequate vitamin D fortification of foods. We estimated that approximately 27,000 cancer deaths were prevented by established fortification policies in 2017, with the potential to prevent an additional 129,000 cancer deaths by implementing vitamin D fortification to the optimal degree in all European countries. More widespread fortification of food could therefore reduce cancer mortality by approximately 10% and thus make a substantial contribution to lowering the burden of cancer mortality in Europe. Uncertainty of those estimations was large, though, as a result of the wide confidence intervals of the underlying studies on vitamin D supplementation and cancer mortality. Nevertheless, even the plausible lower bound of approximately 39,000 cancer deaths annually (34000 in the EU) and 360,000 years of life lost (3128,000 in the EU) being potentially preventable by vitamin D fortification of foods is non-negligible and this potential should be used.

It should be noted that our estimations were done for only one year (2017). Similar calculations could and should be done for more recent time periods as soon as more up-to-date cancer mortality data become available, taking recent changes in fortification policies into account. For example, fortification was limited to margarine in Germany previously [26] but since recently, fortified milk and cereal-, soy-, or almond-based drinks are available. Due to demographic changes and the increasing proportion of elderly people in Europe, the preventive potential of food fortification is expected to increase in future decades.

Vitamin D supplementation is safe in reasonable doses and no side effects are expected at a daily intake of < 3800 IU [27]. The tolerable upper intake level as defined by the European Food Safety Authority (EFSA) and the US Food and Nutrition Board of the Institute of Medicine (FNB/IOM) is even higher, with 4000 IU/day [27], and even daily intake of 5000 IU in fortified bread has been considered safe among nursing home residents [28]. Nevertheless, as vitamin D is fat-soluble and thus, can accumulate in the body, it is theoretically conceivable that some individuals may develop symptoms of hypervitaminosis D (hypercalcemia) such as excessive thirst and urination, fatigue, nausea, and constipation if they additionally supplement vitamin D. In particular, individuals with the (very rare) condition of an abnormally functioning Vitamin D3 24-hydroxylase are at risk of hypercalcemia. To avoid such syndromes, one should ensure adherence to safe upper limits of vitamin D in fortified foods. If fortification becomes common practice, additional vitamin D supplements should only be recommended in cases of diagnosed deficiency or after measurements of serum 25(OH)D and/or calcium levels. Overall, hypervitaminosis D is very rare and has, to our knowledge, not been observed more frequently in fortifying countries than in non-fortifying countries. It is not expected to be of relevant concern for food fortification, which would not aim at such high daily intakes, but rather doses of approximately. 400 IU/day as assumed in our calculations. Nevertheless, to avoid hypervitaminosis D, several precautionary measures should be considered, such as clearly specified upper limits of added vitamin D, based on average dietary habits. In addition, regular measurements of serum 25(OH)D levels (e.g. every 2–3 years) among a representative sample of the population (e.g. *n* = 1,000), along with a questionnaire on intake of supplements and fortified foods should be performed to monitor the efficacy and safety of food fortification. Such surveillance should become part of ongoing health surveillance systems. Furthermore, the frequency of potential − rather rare − adverse effects (e.g. kidney stones) should be monitored (e.g. with health claims data) to ensure that they do not increase in the general population compared to the time prior to food fortification. Finally, we assumed uniform intake of fortified foods. To ensure coverage with fortified foods for the entire population, it would probably be preferable to fortify a wide range of foods, but each food type at a comparably low dose instead of allowing fortification only for a few food types.

A reduction of cancer mortality is not the only expected positive health effect of vitamin D food fortification. RCT based evidence of beneficial health effects of vitamin D supplementation is still limited to a few health outcomes, such as fall and fracture risk [29] (at least when combined with calcium), upper respiratory infections [30], autoimmune disease [31], increased muscle strength [32], improved cognitive function in older adults with Alzheimer’s disease [33], reductions in migraine frequency and severity [34], positive effects on transferrin saturation and iron status [35], and on cardio-metabolic indicators in obese adults [36]. An umbrella review of cohort studies, RCTs and Mendelian randomization studies suggested lower risk of acute respiratory infections, dementia, cognitive decline, and depression mainly in elderly [37]. Decreased systolic blood pressure [38], risk of cardiovascular disease [39] and risk of multiple sclerosis [40] have been found with higher vitamin D status in Mendelian randomization studies. There is suggestive evidence of beneficial effects of vitamin D supplementation for a variety of other disease outcomes such as type 2 diabetes which requires corroboration in further research [41].

An alternative to vitamin D food fortification could be vitamin D supplementation which would enable personalized supply, but would typically reach only a minority of the population. Nevertheless, it might be a relevant first step in countries that have not (yet) established food fortification policies. To our knowledge, there are no studies on the prevalence of vitamin D supplement intake for the majority of European countries. In our sensitivity analysis, we assumed by 10% weaker effects, reflecting a scenario in which a higher than expected proportion of the population already supplemented vitamin D in sufficient doses such that no further cancer mortality reduction can be achieved among them by food fortification. In the UK Biobank as of October 2021, 6% of participants stated (regular) vitamin D intake when they were interviewed (data field 6155). Even though a small share of the population in Germany may have ingested (typically low doses of) vitamin D included in multi-vitamin supplements, there still seems to remain a large unused potential for prevention of cancer deaths.

To our knowledge, our study is the first to comprehensively assess the potential of vitamin D fortification in reducing cancer mortality in Europe. We conducted a comprehensive literature search and contacted relevant health authorities of various countries to obtain information on current fortification policies. The impact of food fortification on cancer mortality was estimated based on a comprehensive investigation of existing food fortification policies and of vitamin D supply by food fortification, and on estimates of the effects of vitamin D supply on cancer mortality which were derived from meta-analyses of large-scale RCTs. We also conducted a sensitivity analysis using smaller effect estimates to incorporate uncertainty in prevalence of vitamin D supplementation.

Our study has several limitations. First, effects of fortification on cancer mortality have not been studied previously and were thus derived from effects of supplementation on cancer mortality and of supplementation and fortification on serum 25(OH)D levels. However, it has been demonstrated that fortification can achieve serum level increases comparable to those for which RCTs have shown significant reductions in cancer mortality [17]. While those RCTs were heterogeneous with respect to administered doses and achieved mortality reduction, inverse associations were observed in all but one study [42] and results are consistent with observational studies. Furthermore, updated results of the VITAL trial found a statistically significant decrease in cancer mortality (HR 0.75, 95% CI 0.59–0.96) when excluding the first two years of follow-up [43]. Also, there was a tendency towards stronger association with higher doses (11% reduction in cancer mortality with 400 IU, 15% with 800 IU, 17% with 2000 IU/day in one study each) [2]. However, current evidence is limited and more data on dose-specific effects of vitamin D would be desirable. In particular, confidence intervals of the effect estimates of vitamin D supplementation on cancer mortality from meta-analyses are still wide (2), and would be compatible with both much higher and much lower reductions in cancer mortality as demonstrated in our sensitivity analyses. Differences in study populations further aggravate comparability of those studies. Second, our study exclusively focused on the effects of food fortification in reducing cancer mortality. For a comprehensive evaluation of food fortification policies, other potentially vitamin D−related health outcomes, as well as expected costs of fortification and corresponding savings (e.g. from prevented cancer deaths and corresponding treatment costs) need to be taken into account. Recent fortification policies were summarized but it was not always possible to obtain information for exactly the year of estimations (2017). However, given that fortification policies typically do not change frequently, the expected impact of this limitation is small. Nevertheless, our findings rely on assumptions regarding the effectiveness of current and hypothetical fortification levels on cancer mortality. Despite evidence on fortification and serum levels, supplementation and serum levels, and supplementation and cancer mortality, direct evidence on an effect of fortification on cancer mortality is not (and most likely will never be) available. Baseline serum vitamin D levels in the different countries were not considered because of a lack of comparable data. However, baseline levels were also not considered in the trials that investigated the impact of vitamin D supplementation on cancer mortality. As such, it is plausible to assume that the assumed 11% cancer mortality reduction rather reflect an average, with stronger reductions in those with below-average serum levels at baseline and weaker reductions in those with above-average serum levels.

The constant decrease in cancer mortality in Finland [44] compared to rather constant mortality rates in Norway [45]—two countries with geographically comparable sun exposure—suggests that the fortification program was actually effective in reducing cancer mortality among the Finish population. Cancer mortality trends are difficult to compare between Norway and Finland, however, due to differences in prevalence of other cancer risk factors such as smoking and alcohol consumption. Implementation of a comprehensive fortification program successfully like the one in Finland might be difficult to achieve in other countries with a more heterogeneous target population with respect to food intake. This underlines the importance of fortifying a wide range of foods.

Notwithstanding the need for further studies to address these important additional points, e.g. quasi-experimental studies using structural breaks (changes in fortification policies), the expected substantial reduction of cancer mortality supports suggestions to promote and implement more widespread use of food fortification in European countries given the high prevalence of vitamin D deficiency and insufficiency [9], particularly in countries in which fortification policies are not established. Obviously, such efforts should supplement, not substitute other urgent efforts in primary prevention of cancer, such as curbing the smoking epidemic or halting and preferably even reversing the rise in overweight and obesity prevalence currently observed in many European countries. Further research is warranted to the estimate effects of vitamin D on cancer mortality by cancer type.

In summary, our study suggests that more widespread vitamin D food fortification policies in European countries might make a major contribution to lowering the burden of cancer deaths in Europe, with the potential to prevent approximately 129,000 additional cancer deaths (more than 113,000 in the EU) and approximately 1.2 million (1 million in the EU) years of life lost per year, corresponding to approximately 9% (10% in the EU) of cancer deaths and years of life lost.

## Supplementary Information

Below is the link to the electronic supplementary material.

Supplementary file1 (DOCX 17 kb)

## Data Availability

The data supporting the findings of this study can be made available via email upon reasonable request. Solely because of a lack of data, we were unable to include all European countries in our calculations. This involves no judgement on any kind of “importance” of included and not included countries. Specifically, we want to express our deepest sympathy for the Ukrainian people. We are shocked by the Russian war on Ukraine and condemn the attacks.

## Abbreviations

RCTs: Randomized clinical trials
IU: International unit
EU: European Union
YLL: Years of life lost
