# Supplementary material for: Vitamin D food fortification in European countries: the underused potential to prevent cancer deaths
Source: Eur J Epidemiol. 2022 May 6;37(4):309–20. doi: 10.1007/s10654-022-00867-4 (PMC9187526; doi:10.1007/s10654-022-00867-4)
Supplement: Supplementary file 1 — Supplementary file1 (DOCX 17 kb) [file 10654_2022_867_MOESM1_ESM.docx]

Supplementary Material of a European Journal of Epidemiology article

**Vitamin D food fortification in European countries: The underused potential to prevent cancer deaths**

**Authors:**

Tobias Niedermaier, Thomas Gredner, Sabine Kuznia, Ben Schöttker, Ute Mons, Jeroen Lakerveld, Wolfgang Ahrens, Hermann Brenner, on behalf of the PEN-Consortium

Content of supplementary material

**Supplementary Table 1.** Life expectancy in the European Union and selected European countries

**Supplementary Table 2.** List of European countries not considered in our calculations because of missing Eurostat data on cancer deaths, together with their population size according to Worldometers.info unless stated otherwise.

**Supplementary Table 1.** Life expectancy in the European Union and selected European countries

| **Country** | **Life expectancy males** | **Life expectancy females** | **Overall life expectancy** | **Reference** |
| --- | --- | --- | --- | --- |
| European Union (27 countries) | 78·2 | 83·6 | 80·9 | Eurostat |
| Austria | 79·3 | 84·1 | 81·7 | Eurostat |
| Belgium | 79·0 | 84·0 | 81·5 | Eurostat |
| Bulgaria | NR | NR | 74·6 | WHO |
| Croatia | NR | NR | 78·3 | WHO |
| Cyprus | NR | NR | 82·7 | WHO |
| Czech Republic | 76·1 | 82·1 | 79·1 | Eurostat |
| Denmark | 79·0 | 82·8 | 80·9 | Eurostat |
| Estonia | NR | NR | 78·0 | WHO |
| Finland | 78·6 | 84·4 | 81·5 | Eurostat |
| France | 79·5 | 85·7 | 82·6 | Eurostat |
| Germany | 78·4 | 83·2 | 80·8 | Eurostat |
| Greece | 78·9 | 84·0 | 81·5 | Eurostat |
| Hungary | NR | NR | 76·3 | WHO |
| Ireland | 79·9 | 83·6 | 81·8 | Eurostat |
| Italy | 81·0 | 85·6 | 83·3 | Eurostat |
| Latvia | NR | NR | 70·9 | WHO |
| Lithuania | NR | NR | 71·3 | WHO |
| Luxembourg | 80·1 | 85·4 | 82·8 | Eurostat |
| Malta | NR | NR | 82·0 | WHO |
| Netherlands | 80·0 | 83·2 | 81·6 | Eurostat |
| Poland | 73·9 | 82·0 | 78·0 | Eurostat |
| Portugal | 78·1 | 84·3 | 81·2 | Eurostat |
| Romania | NR | NR | 75·4 | WHO |
| Slovakia | NR | NR | 77·1 | WHO |
| Slovenia | NR | NR | 81·0 | WHO |
| Spain | 80·5 | 86·3 | 83·4 | Eurostat |
| Sweden | 80·6 | 84·1 | 82·4 | Eurostat |
| Non-EU countries with available data | | | | |
| Iceland | 80·4 | 84·1 | 82·3 | Eurostat |
| Norway | 80·7 | 84·2 | 82·5 | Eurostat |
| Serbia | NR | NR | 75·4 | WHO |
| Switzerland | 81·7 | 85·6 | 83·7 | Eurostat |
| Turkey | 75·4 | 81·0 | 78·2 | Eurostat |
| United Kingdom | 79·4 | 83·0 | 81·2 | Eurostat |

Abbreviations: Eurostat; European Statistical Office; NR, not reported; WHO, World Health Organization.

**Supplementary Table 2.** List of European countries not considered in our calculations because of missing Eurostat data on cancer deaths, together with their population size according to Worldometers.info unless stated otherwise.

| **Country** | **Population** | **Year** |
| --- | --- | --- |
| Ukraine | 44 million | 2020 |
| Belarus | 9·4 million | 2020 |
| Bosnia | 3·3 million | 2020 |
| Albania | 2·9 million | 2020 |
| Moldova | 4·0 million | 2020 |
| Kosovo^1^ | 1·8 million | 2020 |
| Montenegro | 628 thousand | 2020 |
| Monaco | 39 thousand | 2020 |
| Liechtenstein | 38 thousand | 2020 |
| San Marino | 34 thousand | 2020 |
| Vatican City^2^ | 453 | 2019 |
| **Sum** | **~63 million** | **−** |

^1^Data from the World Bank

^2^According to <https://www.vaticanstate.va/it/stato-governo/note-generali/popolazione.html>
